# Oral ketone esters acutely improve myocardial contractility in post-hospitalized COVID-19 patients: A randomized placebo-controlled double-blind crossover study

**DOI:** 10.3389/fnut.2023.1131192

**Published:** 2023-02-09

**Authors:** Helena Zander Wodschow, Filip Søskov Davidovski, Jacob Christensen, Mats Christian Højbjerg Lassen, Kristoffer Grundtvig Skaarup, Hanne Nygaard, Niels Møller, Jørgen Rungby, Tor Biering-Sørensen, Peter Rossing, Nicole Jacqueline Jensen, Jens Christian Laursen

**Affiliations:** ^1^Department of Endocrinology, Copenhagen University Hospital, Bispebjerg and Frederiksberg Hospital, Copenhagen, Denmark; ^2^Department of Cardiology, Copenhagen University Hospital, Gentofte Hospital, Copenhagen, Denmark; ^3^Department of Emergency Medicine, Copenhagen University Hospital, Bispebjerg and Frederiksberg Hospital, Copenhagen, Denmark; ^4^Institute of Clinical Medicine, Aarhus University Hospital, Skejby Hospital, Aarhus, Denmark; ^5^Complications Research, Steno Diabetes Center Copenhagen, Copenhagen, Denmark; ^6^Copenhagen Center for Translational Research, Copenhagen, Denmark; ^7^Department of Clinical Medicine, University of Copenhagen, Copenhagen, Denmark

**Keywords:** ketone bodies, post COVID-19, myocardial metabolism, myocardial contractility, subclinical myocardial injury

## Abstract

**Background:**

COVID-19 is associated with subclinical myocardial injury. Exogenous ketone esters acutely improve left myocardial function in healthy participants and patients with heart failure, but the effects have not been investigated in participants previously hospitalized for COVID-19.

**Methods:**

This is a randomized placebo-controlled double-blind crossover study comparing a single oral ketone ester dose of 395 mg/kg with placebo. Fasting participants were randomized to either placebo in the morning and oral ketone ester in the afternoon or vice versa. Echocardiography was performed immediately after intake of the corresponding treatment. Primary outcome was left ventricular ejection fraction (LVEF). Secondary outcomes were absolute global longitudinal strain (GLS), cardiac output and blood oxygen saturation. Linear mixed effects models were used to assess differences.

**Results:**

We included 12 participants previously hospitalized for COVID-19 with a mean (±SD) age of 60 ± 10 years. The mean time from hospitalization was 18 ± 5 months. Oral ketone esters did not increase LVEF between placebo and oral ketone ester [mean difference: −0.7% (95% CI −4.0 to 2.6%), *p* = 0.66], but increased GLS [1.9% (95% CI: 0.1 to 3.6%), *p* = 0.04] and cardiac output [1.2 L/min (95% CI: −0.1 to 2.4 L/min), *p* = 0.07], although non-significant. The differences in GLS remained significant after adjustment for change in heart rate (*p* = 0.01). There was no difference in blood oxygen saturation. Oral ketone esters increased blood ketones over time (peak level 3.1 ± 4.9 mmol/L, *p* < 0.01). Ketone esters increased blood insulin, c-peptide, and creatinine, and decreased glucose and FFA (all *p* ≤ 0.01) but did not affect glucagon, pro-BNP, or troponin I levels (all *p* > 0.05).

**Conclusion:**

In patients previously hospitalized with COVID-19, a single oral dose of ketone ester had no effect on LVEF, cardiac output or blood oxygen saturation, but increased GLS acutely.

**Clinical trial registration:**

https://clinicaltrials.gov/, identifier NCT04377035.

## Introduction

COVID-19 is associated with myocardial injury (1–3) and has been observed in hospitalized COVID-19 patients as impaired left ventricular- and right ventricular systolic function assessed by echocardiography (4–7), and even in patients that did not require hospitalization assessed by magnetic resonance imaging (8). The degree of impairment in left ventricular function, quantified by absolute global longitudinal strain (GLS), is associated with higher mortality (5, 9, 10) also in the presence of normal left ventricular ejection fraction (LVEF) (11), the recommended parameter for measuring left ventricular systolic function (12). In recovered post-hospitalized COVID-19 patients, GLS, and RVLS are still impaired (13, 14) and an increased risk of cardiovascular disease persists 1 year after infection (15). This indicates a need for improving ventricular function, especially left ventricular function, in post-hospitalized COVID-19 patients.

Several treatment principles for COVID-19 have been suggested. Corticosteroids reduce mortality in patients hospitalized for COVID-19 (16), and reduce the risk of severe disease course (17). Moreover, post-recovery intensive steroid treatment is shown to have positive effects on self-reported long-term symptoms. The antiviral agent, Remdesivir, is widely used in treatment as it shortens recovery time in hospitalized COVID-19 patients (18). Tocilizumab has also been proposed, but was not able to reduce biomarkers of cardiac injury during hospitalization (19). One study on cardiovascular rehabilitation concerning exercise in post-hospitalized COVID-19 patients has shown positive results on physical performance (20). This study did, however, not include objective assessment of cardiac function, and though treatment of acute COVID-19 infection is extensively covered, studies on treatment of cardiovascular sequela and prognostic prevention are lacking. New interventions are needed.

Intravenous ketone body infusion acutely increases LVEF, GLS, and cardiac output in patients with heart failure with reduced ejection fraction (21). To our knowledge, the effects of ketone bodies on myocardial function have never been investigated with an oral intervention in subjects with subclinical myocardial dysfunction, as can be seen following COVID-19. The aim of the present study was to assess the acute effects of oral ketone ester on LVEF, GLS, cardiac output, and peripheral blood oxygen saturation in post-hospitalized COVID-19 patients with the hypothesis that these measures can be improved. To gain mechanistic insight, we exploratively investigated the effects of additional echocardiographic parameters, blood biochemistry, and vital values.

## Materials and methods

### Study design

This randomized, placebo-controlled, double-blind cross-over acute intervention study was a sub-study of the ECHOVID-19 study (4, 22), a prospective longitudinal study investigating echocardiographic parameters in adults hospitalized with COVID-19. Participants were randomly allocated in a 1:1 ratio to either placebo in the morning and oral ketone ester in the afternoon (sequence A) or vice versa (sequence B). The study was conducted from May to December 2021 and was carried out at the Department of Endocrinology, Copenhagen University Hospital – Bispebjerg and Frederiksberg Hospital.

### Participants

Twelve post-hospitalized COVID-19 patients were enrolled from the ECHOVID-19 study. Inclusion criteria for ECHOVID-19 were adults hospitalized in hospitals of the Capital- and Zealand regions with a laboratory confirmed diagnosis of COVID-19. Exclusion criteria for ECHOVID-19 were patients unable to understand and sign informed consent, or too sick to cooperate. Additional exclusion criteria for the present study were a diagnosis of chronic obstructive pulmonary disease or asthma, active treatment with sodium-glucose 2 inhibitors, eGFR < 15 ml/min/1.73 m^2^ or insulin-dependent diabetes. All participants gave informed consent. The study was performed in accordance with the Second Declaration of Helsinki and approved by the regional ethics board (H-20021500). The ECHOVID-19 study is registered at Clinicaltrials.gov (NCT04377035) and the present sub-study likewise (NCT04573764).

### Study procedures

Participants arrived between 7.45 and 8.00 am after an overnight fast and remained fasting until the end of the visit. For the intervention, a single oral ketone ester (KetoneAid Inc., Falls Church, VA) dose of 395 mg/kg bodyweight, containing primary the D-isoform, was compared with taste- and volume-matched placebo provided by the same company. The intervention (ketone esters and placebo) was consumed using a sipping method over 60 min, the purpose of which was to promote steady state and increase the timespan that ketones would be elevated (23): the first third of the intervention was consumed at baseline (t0), the second third after 20 min (t20), and the last third after 60 min (t60). To prevent carry-over, the design included a washout period of 2 h leading to the last part of the morning intervention being ingested 3.5 h before commencing the afternoon intervention. A bedside echocardiography was performed immediately after the last sip of the corresponding treatment (Supplementary Figure 1). Blood samples were taken every half hour from t0 to the end of the intervention at t150. Vitals were measured before blood sampling at t0 and t60 on the same arm of the subject: blood pressure and pulse were measured using a standard hospital cuff, peripheral blood oxygen saturation using a standard finger pulse oximeter. A continuous infusion of glucose (10% solution, 50 mM potassium-chloride) was initiated before baseline at 50 ml/h to maintain euglycemia.

Characteristics and COVID-19 complications were achieved through patient journals and information on medicine through self-report. BMI was calculated on the day of the trial.

### Outcomes

The primary outcome was left ventricular ejection fraction (LVEF). The secondary outcomes were absolute global longitudinal strain (GLS), cardiac output, and peripheral blood oxygen saturation. Exploratory outcomes were additional echocardiographic measures of systolic- and diastolic function, blood sample biochemistry, and vital values.

#### Echocardiography

Echocardiography was performed bedside with a portable Vivid IQ Ultrasound System (GE Healthcare, Horten, Norway). Examinations were performed according to a pre-determined protocol by two trained sonographers who were blinded to the intervention. The recordings were analyzed offline using commercially available post-processing software (ECHOPAC Version 203, GE Vingmed Ultrasound AS). Each parameter was analyzed separately by one of two trained investigators blinded to all clinical information. All echocardiographic measurements were performed and analyzed according to existing guidelines (12). Abnormal echocardiographic findings were assessed from the echocardiography performed during the placebo-intervention.

Left ventricular volumes and LVEF were measured using Simpson’s biplane method. For GLS, two-dimensional speckle tracking was performed offline in the three apical views (four-chamber, two-chamber, and three-chamber view) of the left ventricle. Frame rate was optimized for speckle tracking analysis. The myocardial wall was traced with a semi-automatic function and manually adjusted in case of inaccurate tracing. GLS was measured by dividing each of the three projections into six segments and integrating the segments into a global 18-segment model of the LV. Global values were obtained by averaging the strain values of all included segments. Segments deemed untraceable by the investigators were excluded. Cardiac output was measured from left ventricular outflow tract. TAPSE was measured by M-mode in the apical four-chamber view. Peak mitral inflow velocity (E-wave, A-wave, and E/A ratio) and deceleration time of E were measured with pulsed-wave Doppler in the four-chamber view. Color tissue Doppler velocities (a’, e’, and s’) were measured at the septal and lateral wall of the mitral annulus in the apical four-chamber view. E-wave was indexed to e’ to estimate E/e’. Left atrial volume was measured by the area-length method in the apical two-chamber and four-chamber view. Abnormal LVEF (<52% for males and <54% for females), GLS (<16%) and TAPSE (<1.7 cm) were defined according to existing guidelines (12).

#### Blood samples

Blood samples for glucose and BHB analyses were collected at six timepoints every half hour from t0-t150 for the morning and afternoon periods, respectively, while samples for free fatty acids (FFA), insulin, troponin I, creatinine, pro-brain natriuretic peptide (pro-BNP) and glucagon were collected at baseline (morning t0), t60 morning and t60 afternoon.

FFA, creatinine, troponin T, pro-BNP, glucose and BHB were stored at −20 degrees Celsius, and insulin and glucagon were stored at −80 degrees Celsius until analysis. FFA and Insulin were analyzed from serum and the rest from EDTA or Li/hep plasma. Total BHB was analyzed from whole blood using hydrophilic interaction liquid chromatography tandem mass spectrometry. Insulin and glucagon were measured with ELISA (Mercodia, Sweden). FFA was quantified by *in vitro* enzymatic colorimetric method assay (Trichem, Denmark). Pro-BNP was analyzed by chemiluminescent microparticle immunoassay on an Abbott Architect i2000SR (Abbott, Germany), and c-peptide by direct chemiluminescent immunoassay (Atellica IM, USA). Troponin I was analyzed by an immunoassay kit (Cobas; Roche Diagnostics GmbH, Germany). Glucose and creatinine were analyzed by enzymatic absorption photometry (Atellica CH, USA). All analyses were performed by blinded personnel.

### Statistical methods and sample size calculation

Data are reported as means ± standard deviation (SD) or, if skewed distributions, as medians with interquartile range [interquartile range (IQR)]. GLS is reported as the absolute value.

A *general linear mixed model* (R package “LMMstar”) was used to estimate the difference between intervention and placebo. Time (morning versus afternoon) and intervention (placebo versus ketone esters) were used as fixed effects. Participant ID was used as random effect. A *p*-value of <0.05 was considered significant. For the echocardiographic outcomes, significant associations were further adjusted for change in heart rate, as this might affect results. Echocardiographic outcomes were adjusted for sequence *post hoc*. Vitals and blood values were adjusted for baseline values. Comparison between the ECHOVID-19 follow-up cohort and participants in the present study were calculated by *t*-test and Fisher’s exact test. Percentage is calculated from number of measured valuables (not including missing values). All statistical analyses and graphical illustrations were carried out using R Statistical Software [R version 4.1.0; R Core Team (24)].

The randomization list was generated by an unrelated study nurse and was performed in fixed blocks of six (allocating three participants to each sequence ketone-placebo vs. placebo-ketone) using the “blockrand” package in R. Personnel and study participants involved in the study were blinded to the randomization code until the end of the last participant’s last visit.

The power calculation was based on a study demonstrating improved LVEF in patients with heart failure with reduced ejection fraction after continuous intravenous BHB infusion where mean ± SD LVEF changed from 35 ± 7% to 43 ± 9% (21). Assuming oral ketone esters would change LVEF 8% with a mean SD of 8%, the sample size required to demonstrate a significant effect with a power of 80% and type 1 error of 5% was 10 participants. To account for missing data and errors, 12 participants were included. Power calculation was performed using the power statement implemented in the SAS enterprise software 7.1 (SAS Institute, Cary, NC, USA).

### Protocol changes

The study was initially meant to include twelve hospitalized patients with COVID-19 recruited from the ECHOVID-19 trial but was changed to include post-hospitalized patients because the in-hospital setting proved unfeasible for the study procedures. Blood gas analyses and urine creatinine clearance were study outcomes originally but turned out to be unfeasible and removed.

## Results

### Study participants and intervention

**FIGURE 1:**
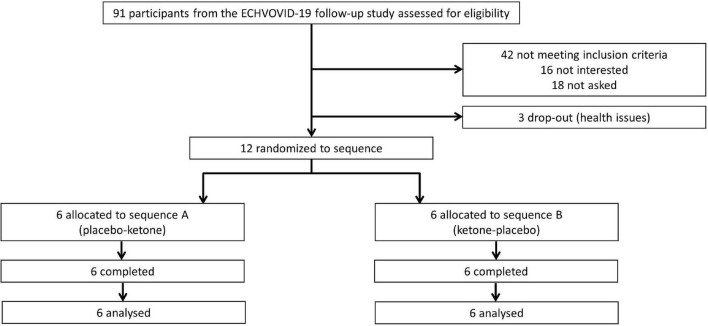
Participant flow diagram. Three dropped out due to personal health issues (one broken foot, one had progression in cancer, one due to worsening of general health).

The ECHOVID-19-study counted 215 patients of whom 43 did not survive. From the remaining 172 participants, 91 participated in a follow-up visit 2–3 months after hospitalization and were assessed for eligibility for this sub-study. Forty-two did not meet the additional criteria for participation, 16 were not interested, 3 dropped-out before randomization due to personal health issues, and 18 were never asked because 12 participants had completed the study (Figure 1).

We included 12 participants (5 women) with a mean ± SD age of 60 ± 10 years. All women were by chance randomized to sequence B. The mean time from hospitalization was 18 ± 5 months, 5 (42%) had a diagnosis of post-COVID-19 sequela, of whom 2 had radiological signs of fibrosis (without self-reported effect on daily life), and 3 had post-viral mental fatigue or self-reported cognitive decline. All participants were Caucasians and non-smokers (Table 1). Compared to ECHOVID-19, participants included in this study had higher systolic blood pressure and lower heart rate compared with those not included from the follow-up cohort, but where otherwise comparable (Supplementary Table 1). The participants with GLS < 16% during hospitalization (*n* = 3) also had a GLS < 16% in the present study.

**TABLE 1:** Clinical characteristics.

	Placebo-ketone (A)	Ketone-placebo (B)	Total
*n*	6	6	12
**Characteristics**			
Age (years)	63 ± 5	55 ± 8	59 ± 8
Male sex, *n* (%)	6 (100%)	1 (17%)	7 (58%)
BMI (kg/m^2^)	26.0 ± 4.2	28.5 ± 7.9	27.3 ± 6.2
Smoking	0	0	0
Hypertension, *n* (%)	2 (33%)	1 (17%)	3 (25%)
Hyperlipidemia, *n* (%)	4 (66%)	3 (50%)	7 (58%)
Prevalent heart failure, *n* (%)	0	0	0
Ischemic heart disease, *n* (%)	1 (17%)	1 (17%)	2 (17%)
**COVID-19 complications**			
Time from hospitalization (months)	18 ± 4	17 ± 7	17 ± 5
Length of hospitalization (days)	9 [5; 11]	6 [2; 19]	8 [4; 14]
Admission to intensive care unit, *n* (%)	1 (17%)	2 (33%)	3 (25%)
Acute respiratory distress syndrome, *n* (%)	0	1 (17%)	1 (8%)
Venous thromboembolic event, *n* (%)	1 (17%)	0	1 (8%)
Diagnosis of post-COVID-19 sequela, *n* (%)	2 (33%)	3 (50%)	5 (42%)
**Medicine, *n* (%)**			
Direct oral anticoagulants	3 (50%)	0	3 (25%)
Acetylsalicylic acid	0	3 (50%)	3 (25%)
Lipid-lowering agents	4 (67%)	2 (33%)	6 (50%)
Beta-blocker	3 (50%)	1 (17%)	4 (33%)
Calcium-antagonist	1 (17%)	0	1 (8%)
ACE-inhibitor	1 (17%)	0	1 (8%)
**Baseline values**			
Systolic blood pressure (mmHg)	145 ± 13	128 ± 10	136 ± 14
Diastolic blood pressure (mmHg)	85 ± 12	79 ± 6	82 ± 10
Heart rate (bpm)	58 ± 7	65 ± 9	62 ± 9
Oxygen saturation (%)	99.1 ± 0.8	98.3 ± 1.1	98.9 ± 0.9
**Cardiac involvement, *n* (%)**			
Abnormal LVEF	1 (17%)	1 (17%)	2 (17%)
Abnormal GLS	1 (17%)	3 (50%)	4 (33%)
Abnormal TAPSE	0	0	0

Data for all participants are divided by sequence and presented as either mean ± SD, median [IQR], and *n* (%). Abnormal echocardiographic findings were assessed from the echocardiography performed during the placebo-intervention. ACE, angiotensin-converting enzyme; BMI, body mass index; bpm, beats per minute; GLS, global longitudinal strain; LVEF, left ventricular ejection fraction; TAPSE, tricuspid annular plane systolic excursion.

**FIGURE 2:**
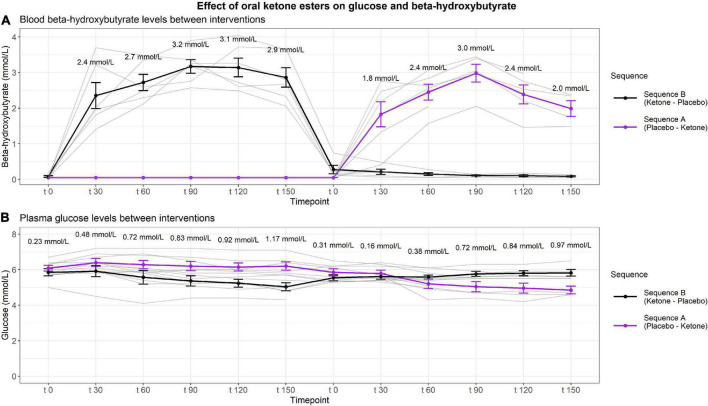
Effect of ketone esters on BHB and glucose levels. The figure depicts the changes in BHB levels assessed from whole blood and shows the average peak BHB-levels at the different timepoints **(A)** and depicts the absolute changes in glucose assessed from plasma **(B)** between interventions given as mean ± SEM. Data was analyzed by general linear mixed model adjusted for time of day, t0 and interaction between timepoints and intervention. Blood samples were collected at six timepoints every half hour from t0 to t150 for morning and afternoon, respectively.

Total blood BHB increased with ketone esters vs. placebo over time (*p* < 0.01) and reached the highest mean levels at t90 (3.1 ± 4.9 mmol/L) (Figure 2A). Mean plasma glucose decreased between 0.53 and 0.97 mmol/l from t60-t150 with ketone esters vs. placebo (*p* = 0.01) (Figure 2B).

### Effect of ketone esters on echocardiographic parameters

**FIGURE 3:**
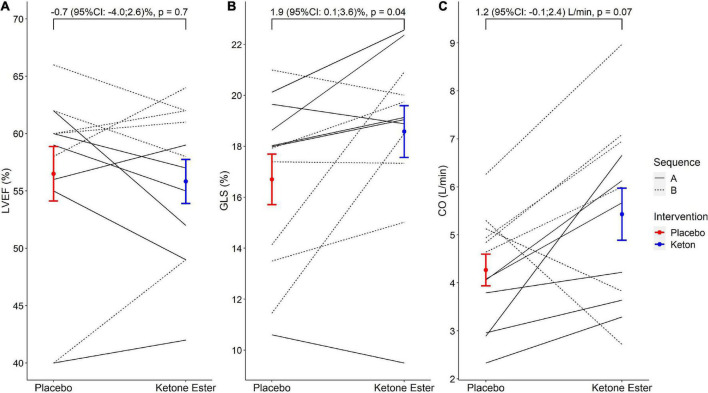
The effects of oral ketone esters on LVEF, GLS, and cardiac output. The figure depicts the absolute changes (mean ± SEM) between placebo and oral ketone esters for LVEF **(A)**, GLS **(B)**, and cardiac output **(C)**, respectively. The brackets report unadjusted mean differences (95% CI) with *p*-values. LVEF, left ventricular ejection fraction; GLS, absolute global longitudinal strain.

Compared with placebo, oral ketone esters had no effect on LVEF with a mean difference of −0.7 (95% CI: −4.0 to 2.6)%, *p* = 0.66 (Figure 3A). GLS was significantly improved after oral ketone esters compared with placebo, with a mean GLS of 16.7 ± 3.4% after placebo and 18.6 ± 3.5% after oral ketone esters. This corresponded to a mean difference of 1.9 (95% CI: 0.1 to 3.6)%, *p* = 0.04 (Figure 3B). The difference remained significant after adjustment for change in heart rate (*p* < 0.01). Cardiac output was increased, although not significantly, by oral ketone esters, with a mean cardiac output of 4.3 ± 1.1 L/min after placebo and 5.4 ± 1.9 after oral ketone esters which correspond to a mean difference of 1.2 (95% CI: −0.1 to 2.4) L/min, *p* = 0.07 (Figure 3C). Stroke volume increased with 11 ml with ketones versus placebo, but the change was not significant (*p* = 0.2). Right ventricular systolic function did not improve as assessed by TAPSE (*p* > 0.05), nor did diastolic function as assessed by mitral valve (MV) E/e’ or MV E/A ratio or MV E-wave deceleration time (*p* > 0.05) (Table 2). When adjusting for sequence, cardiac output and cardiac index increased significantly (both *p* < 0.05).

**TABLE 2:** Differences in echocardiographic parameters between oral ketone esters and placebo.

Variable	Placebo	Ketone	Mean difference [CI]	*P-*value	Adjusted *P-*value
**Left ventricular systolic function**					
Left ventricular ejection fraction, (%)	57 ± 8	56 ± 7	−0.7 [−4.0; 2.6]	*p* = 0.66	
Absolute global longitudinal strain, (%)	16.7 ± 3.4	18.6 ± 3.5	1.9 [0.1; 3.7]	*p* = 0.04	*p* < 0.01
Cardiac output, (L/min)	4.3 ± 1.1	5.4 ± 1.9	1.2 [−0.1; 2.4]	*p* = 0.07	
Cardiac index, (L/min/m^2^)	2.1 ± 0.7	2.7 ± 1.1	0.6 [−0.1; 1.2]	*p* = 0.08	
Left ventricular end-diastolic volume, (mL)	94.0 ± 22.6	88.1 ± 25.9	−5.9 [−15.7; 3.9]	*p* = 0.21	
Left ventricular end-systolic volume, (mL)	41.0 ± 12.1	38.9 ± 12.6	−2.1 [−7.2; 3.0]	*p* = 0.38	
Left atrial end-systolic volume, (mL)	45.3 ± 16.6	47.9 ± 14.6	2.8 [−10.0; 15.5]	*p* = 0.63	
Stroke volume, (mL)	71.3 ± 12.4	82.0 ± 29.0	10.7 [−6.5; 28.0]	*p* = 0.20	
**Diastolic function**					
MV E/e’	7.1 ± 1.3	6.9 ± 1.4	−0.3 [−1.3; 0.8]	*p* = 0.55	
MV E/A ratio	1.1 ± 0.3	1.0 ± 0.3	−0.1 [−0.2; 0.0]	*p* = 0.11	
MV E-wave deceleration time, ms	275 ± 89	238 ± 92	−37 [−85; 11]	*p* = 0.12	
**Right ventricular function**					
TAPSE, (mm)	2.4 ± 0.6	2.4 ± 0.6	0.05 [−0.4; 0.4]	*p* = 0.80	

Data are mean ± SD and mean differences with 95% CI. Data are analyzed by general linear mixed model; significant differences were adjusted for change in heart rate. There was one missing datapoint for left atrial end-systolic volume, three for MV E/e’, and one for TAPSE. Right ventricular longitudinal strain was excluded (four missing datapoints and three outliers). MV, mitral valve; TAPSE, tricuspid annular plane systolic excursion.

When stratified according to normal (>16%) and abnormal GLS (<16%), GLS was increased on average by 3.6% for participants with abnormal GLS and 0.9% for participants with normal GLS, though insignificant (*p* for interaction = 0.2). Ketone esters elevated GLS to above the cut-off value of 16% for 2 out of 4 participants with abnormal GLS, yet results were insignificant (*p* = 0.6) (Supplementary Figure 2). We found no association between blood levels of BHB and GLS increase with an estimate of −0.001 (95% CI: −0.004 to 0.001)%, *p* = 0.3.

### Effect of ketone esters on blood values and vitals

Oral ketone esters increased blood insulin by 36 (95% CI: 21 to 50) pmol/L, c-peptide with 265 (95% CI: 156 to 374) pmol/L, and creatinine with 2 (95% CI: 1 to 4) μmol/L compared with placebo, and decreased FFA with 0.26 (95% CI: −0.33 to −0.18) mmol/L. Ketone esters did not affect glucagon, pro-BNP, or troponin I levels (all *p* > 0.05) (Table 3).

**TABLE 3:** Effect of ketone esters on blood values and vitals.

Variable	Baseline	Placebo	Ketone	Mean difference [CI]	*P-*value
**Blood values**					
Insulin, (pmol/L)	48 ± 23	51 ± 26	87 ± 40	36 [21; 50]	*p* < 0.01
C-peptide, (pmol/L)	696 ± 226	760 ± 290	1025 ± 355	265 [156; 374]	*p* < 0.01
Free fatty acids, (mmol/L)	0.42 ± 0.20	0.35 ± 0.16	0.09 ± 0.05	−0.26 [−0.33; −0.18]	*p* < 0.01
Glucagon, (pmol/L)	8.2 ± 2.8	6.6 ± 2.1	5.8 ± 2.2	−0.8 [−2.0; 0.4]	*p* = 0.16
Creatinin, (μmol/L)	78 ± 20	73 ± 22	75 ± 19	2 [1; 4]	*p* < 0.01
pro-BNP, (pmol/L)	10.1 [6.0; 27.8]	11.9 [6.4; 30.6]	11.2 [7.5; 30.6]	−0.2 [−3.1; 2.6]	*p* = 0.87
Troponin I, (ng/L)	4.5 [3.0; 5.8]	4.5 [3.8; 6.5]	5.0 [3.8; 6.0]	0 [−0.4; 0.4]	*p* = 1.00
**Vitals**					
Heart rate, (bpm)	61 ± 9	59 ± 9	66 ± 7	10 [7.4; 13.2]	*p* < 0.01
Systolic blood pressure, (mmHg)	135 ± 15	134 ± 14	133 ± 16	−2.8 [−8.1; 2.5]	*p* = 0.26
Diastolic blood pressure, (mmHg)	81 ± 9	81 ± 10	78 ± 11	−3.4 [−5.1; −1.8]	*p* < 0.01
Saturation, (%)	98.9 ± 0.9	98.7 ± 0.5	98.7 ± 1.4	0.3 [−0.2; 0.8]	*p* = 0.19

Data are mean ± SD or median [IQR]. Data are analyzed by general linear mixed model. Blood samples were taken at baseline (morning t0) and at the end of each intervention (t60). Mean difference and 95% CI were adjusted for baseline values and time of day. Vitals were measured at t0 and t60 for each intervention. Mean difference and 95% CI were adjusted for the respective t0 value and time of day. The reported baseline values for vitals are the average of both t0 measurements. Bpm, beats per minute.

Oral ketone ester increased heart rate 10 (95% CI: 7 to 13) bpm. Ketone esters had no effect on systolic blood pressure (*p* > 0.05) but lowered diastolic blood pressure by 3.4 (95% CI: −5.1 to −1.8) mmHg. Ketone esters had no effect on peripheral blood oxygen saturation (*p* > 0.05).

## Discussion

In this study, we found no effect of oral ketone esters on LVEF in post-hospitalized COVID-19 patients, but we are the first to demonstrate that a single oral dose of ketone esters could increase GLS acutely compared with placebo in post-hospitalized COVID-19 patients. Oral ketone esters had no effect on cardiac output or peripheral blood oxygen saturation, but increased heart rate and lowered diastolic blood pressure. Blood ketones, insulin, c-peptide, and creatinine were increased while glucose and free fatty acids were decreased compared with placebo.

### Improvement in cardiac function following treatment with oral ketone esters

In hospitalized COVID-19 patients, reduced GLS is independently associated with death (4). In patients with heart failure with reduced ejection fraction (LVEF: 35 ± 7%), intravenous infusion of ketone salts to 3.3 mmol/L has been shown to improve left ventricular systolic function assessed as an increase in LVEF by 8%, GLS by 2%, and cardiac output by 2 L/min. Right ventricular systolic function was also improved assessed as an increase in TAPSE by 0.2 cm after ketones (21). In the present study on patients with suspected COVID-19-induced myocardial damage, we similarly found significant improvements in GLS at comparable BHB levels (2.5–3.1 mmol/L), however, in a population with only subclinical or normal cardiac function as assessed by GLS and, importantly, using an oral intervention which is a more feasible approach to achieve ketosis. We did not observe an increase in LVEF, which might be explained by our study participants having a normal baseline LVEF (57% ± 8). In patients with heart failure, intravenous ketone esters increase cardiac output in a dose-dependent manner (21), and in a study on healthy participants, a single oral dose of ketone esters reaching higher BHB levels than in the present study [median blood-BHB concentration: 3.23 mmol/L (IQR: 2.40–4.97)] LVEF was increased by 3.1%, GLS by 2.0%, and right ventricle S’ by 1.1 cm (25). Myocardial BHB utilization is furthermore increased in proportion to blood ketone levels (26), indicating that our failure to report an increase in LVEF and cardiac output could also be dose dependent. On the contrary, we were not able to demonstrate an association between blood levels of BHB and a GLS increase, which does not support a dose-response relationship. This could, however, be due to the small sample size. In the present study we did not find an improvement in right ventricular systolic function as assessed by TAPSE and right ventricular longitudinal strain was excluded due to lacking data. The insignificant results of TAPSE are probably due to the same mechanisms as for LVEF. Diastolic function was not found to be impaired compared with controls in a study with hospitalized COVID-19 patients (4) but was, however, impaired in hospitalized COVID-19 patients with simultaneous elevated high-sensitive troponin levels or classified as being severely ill (6, 7). In the present study we did not find any effect of ketone esters on diastolic function as assess by MV E/A ratio, MV E/e’, or MV E-wave deceleration time. To our knowledge, no effect of ketone esters on diastolic function has previously been reported (21, 25). With oral ketone esters elevating GLS to 18.6 ± 3.5% in the present study, we propose that oral ketone esters could normalize impaired GLS in in patients with asymptomatic subclinical myocardial dysfunction. In fact, ketones esters did indeed raise GLS for 2 out of 4 of the participants with GLS < 16% to within the normal range. Although insignificant, probably due to the small sample size, this suggests a larger effect in those with abnormal GLS. Interestingly, endogenous BHB concentrations rise in response to the degree of cardiac dysfunction (27), and one dose of oral ketone esters enhanced myocardial BHB extraction in patients with heart failure with reduced ejection fraction compared with controls and correlates with the degree of cardiac dysfunction (28). These findings support the hypothesis that increased BHB utilization in the failing heart could be an adaptive mechanism. The effects of BHB on different degrees of myocardial malfunction from subclinical lowered myocardial function to manifest heart failure should be explored in future studies.

### Metabolic changes in heart failure

Both right and left ventricular function are impaired in patients formerly hospitalized for COVID-19 (13, 14). The healthy human heart utilizes both fatty acids, glucose, and ketone bodies (29, 30), but prefers fatty acids over glucose. In heart failure, BHB utilization along with the rate-limiting enzyme in ketone oxidation are increased (26, 31), which could be a beneficial adaptive mechanism, since ketone oxidation is highly energy efficient compared to fatty acids (32), and is utilized in proportion to blood-levels (26), replace myocardial glucose uptake and increase myocardial blood flow (33), and can occur independent of insulin (34). Sodium-glucose cotransporter 2 (SGLT2) inhibitors induce ketosis in patients with diabetes mellitus type 2 (5.6 ± 6.0 μmol/L when fasting and 3.3 ± 3.8 μmol/L when fed), but not in patients without diabetes (35) and reduce the risk of cardiovascular death and hospitalization for heart failure in both groups (36, 37). It has been suggested that ketone bodies might have a direct metabolic cardioprotective effect (38). In patients with T2D without heart disease, treatment with SGLT2 inhibitors increase GLS but only in the group with subclinical myocardial dysfunction (GLS < 16.5%) (39). This supports the proposed hypothesis that the effect of ketone esters on myocardial contractility depends on the degree of cardiac dysfunction. Our data indicates an improvement in left ventricular systolic function following consumption of ketone esters and thus supports the hypothesis that BHB is a competitive, efficient, and thrifty substrate for the struggling heart by increasing myocardial tissue energy availability.

In the present study we demonstrated a decrease in free fatty acids and glucose, and an increase in insulin-production and plasma-creatinine under constant glucose infusion. Exogenous ketosis decreases free fatty acids and blood-glucose and increases plasma-insulin (23, 33, 40) in contrast to endogenous ketosis which is characterized by low levels of insulin and glucose, and high free fatty acids (23). High blood ketone levels have been shown to inhibit lipolysis by negative feedback, and lower glucose levels through inhibiting gluconeogenesis (41) and by increasing peripheral glucose uptake and glycogen synthesis in muscles (40). Accumulation of toxic lipid intermediates is associated with myocardial insulin-resistance in heart failure, and active unloading leads to improved cardiac insulin signaling (42). In theory, ketone bodies might therefore be cardioprotective by inhibition of free fatty acids. To our knowledge, there are no studies testing the effect of exogenous ketone esters on toxic lipid accumulation in the heart, and studies on associations between post-infectious sequela and ketosis are severely lacking.

Besides their metabolic effects, ketone bodies also serve as signaling molecules and have been shown to reduce oxidative stress and inflammation in several tissues (43). COVID-19 virus infection is known to induce an inflammatory response and even months after infection, myocardial inflammation can be detected in some patients (44). It could therefore be speculated that the improved cardiac function found following ketone treatment is partly related to anti-inflammatory properties of ketone bodies.

Ketone esters have been shown to increase heart rate and decrease systemic vascular resistance while having no effect on blood-pressure (21). Similar findings were observed in the present study.

### Implications and further studies

The present study is the first to demonstrate that oral ketone esters acutely improve left ventricular systolic function in post-hospitalized COVID-19 patients and that oral ketone esters may represent a novel treatment principle in combatting the impending rise in COVID-19 sequela following the pandemic and other conditions with subclinical lowered myocardial function. Our study supports the hypothesis that increased BHB utilization in the failing heart is an adaptive mechanism and might help explain the cardioprotective effects of SGLT2 inhibitors. Studies are needed to investigate if ketone bodies have mechanisms other than increasing energy availability such as possible effects on toxic lipid accumulation in the heart, and if the effect of ketone bodies increases with the degree of heart decompensation. Studies on the chronic effects of ketone bodies are warranted.

### Strengths and limitations

A limitation is the relatively small sample size, although we did have power to demonstrate effects on GLS. The sample size was calculated from a population with reduced ejection fraction which theoretically should have a greater effect of ketone esters than in the cohort of the present study. The study might therefore be underpowered which could explain the negative results on the primary outcome. Participants in the present study had significantly higher systolic blood pressure and lower heart rate compared with the follow-up cohort though they were comparable on all other parameters including no difference in odds for hypertension, ischemic heart disease or prevalent heart failure. The cohort in the present study was therefore deemed representative for participants in the main study. Participants were not stratified for the outcome of previous echo-examinations and the below-expected values of GLS are assumed to be COVID-19-related, just as patients recovered from COVID-19 have significantly impaired left and right ventricular function compared to matched controls (14). However, there is no definite proof that COVID-19 and subclinical systolic dysfunction are related in our study population and therefore, the results of the present study should be interpreted as the effect of oral ketone esters on patients with asymptomatic subclinical myocardial dysfunction and not specifically in patients previously hospitalized with COVID-19. Five outcomes were significant for time: insulin, c-peptide and creatinine were higher in the morning while heart rate and cardiac output were higher in the afternoon. The observed periodic effect was accommodated by adjusting for time. A possible carry-over effect was accommodated by including a wash-out period of 2 h leading to the last part of the morning intervention being ingested 4 h before commencing the afternoon intervention. For orally ingested ketone esters, the time taken to reach the maximal concentration is approximately 1-h, coinciding with the echocardiography. Elimination is non-linear and follows first order elimination kinetics, eliminating approximately 130 mmol/min by peripheral oxidation (23). Hence, the design should hereby have made any carry-over effect negligible. Another limitation was the unequal distribution of sex between the two sequences with all women by chance being randomized to sequence B and with 50% being characterized by abnormal GLS opposed to only 17% of sequence A (Table 1). This distribution was accommodated by the paired design. When adjusting for sequence, cardiac output and cardiac index became significant (both *p* < 0.05) while having no impact on the other results. This indicates that the observed effect of oral ketone esters on cardiac function was not driven by differences in the sequence groups.

Strengths of the study include that both interventions were conducted on the same day making the circumstances for the individual participant as identical as possible and therefore avoiding day-to-day variations. The study was performed according to standardized procedures.

## Conclusion

In patients previously admitted with COVID-19, moderate ketosis achieved by a single oral dose of ketone esters increased GLS acutely and independently of heart rate but had no effect on LVEF, cardiac output or blood oxygen saturation. Our study suggests a direct improvement in left ventricular myocardial function in subjects previously hospitalized for COVID-19. Oral ketone esters may represent a novel treatment principle in combatting the impending rise in COVID-19 sequela following the pandemic and other conditions with subclinical cardiac injury.

## Data availability statement

The original contributions presented in this study are included in the article/Supplementary material, further inquiries can be directed to the corresponding author.

## Ethics statement

The studies involving human participants were reviewed and approved by the Danish Ethical Committee on Health Research Ethics, Copenhagen, Denmark. The patients/participants provided their written informed consent to participate in this study.

## Author contributions

HW: formal analysis, investigation, resources, data curation, writing – original draft, writing – review and editing, visualization, and project administration. FD: software, data curation, and writing – review and editing. JC: software, investigation, data curation, and writing – review and editing. ML, KS, and HN: writing – review and editing. NM: conceptualization, validation, and writing – review and editing. JR and PR: conceptualization, validation, resources, writing – review and editing, and supervision. TB-S: conceptualization, validation, resources, and writing – review and editing. NJ: conceptualization, methodology, validation, resources, writing – review and editing, and funding acquisition. JL: conceptualization, methodology, validation, investigation, resources, data curation, writing – review and editing, and funding acquisition. All authors contributed to the article and approved the submitted version.
