# Supplementary figures and images for: Oral ketone esters acutely improve myocardial contractility in post-hospitalized COVID-19 patients: A randomized placebo-controlled double-blind crossover study
Source: Front Nutr. 2023 Feb 9;10:1131192. doi: 10.3389/fnut.2023.1131192 (PMC9947401; doi:10.3389/fnut.2023.1131192)

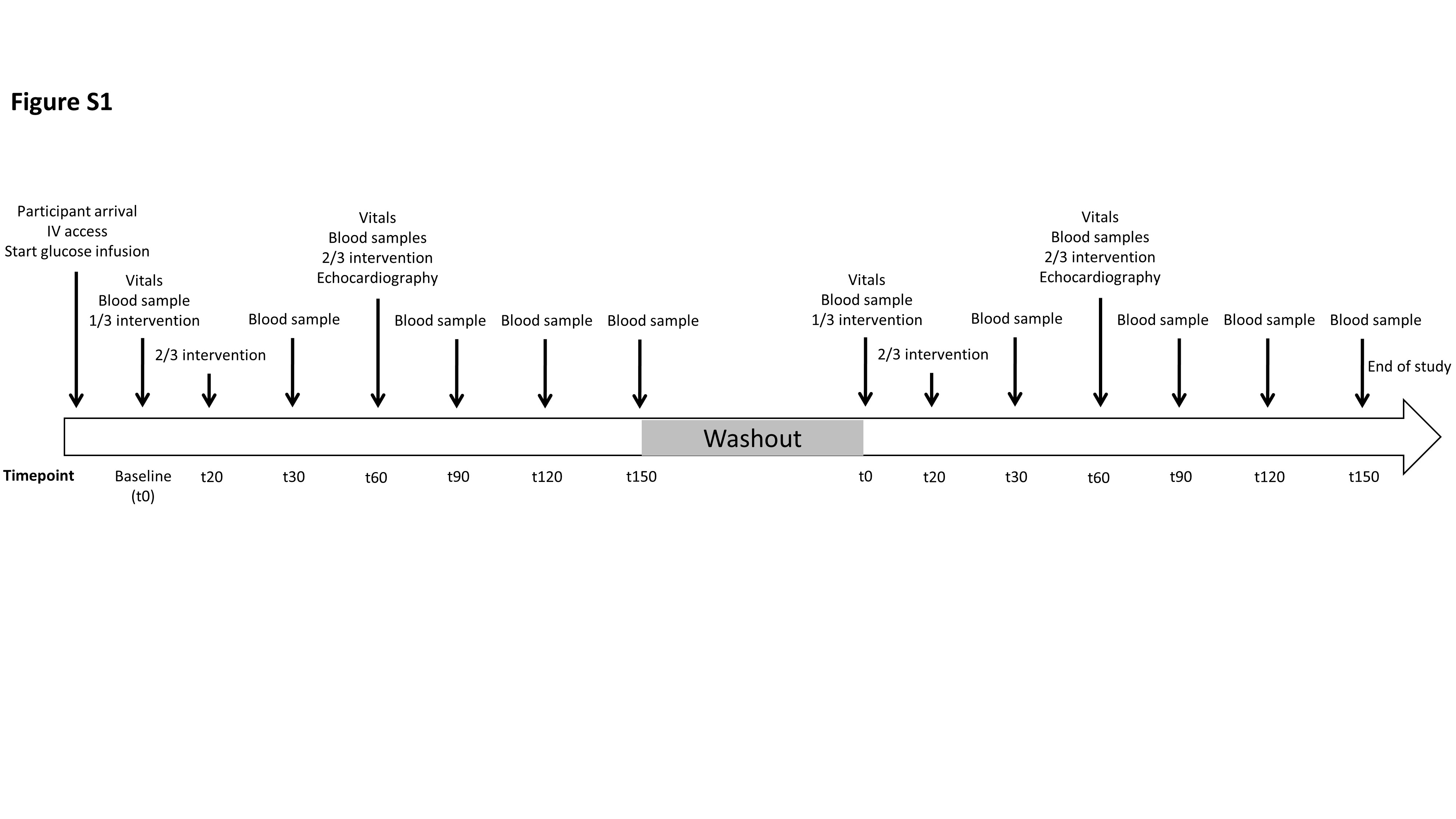

Supplement: Supplementary file 1 [file Image_1.JPEG]

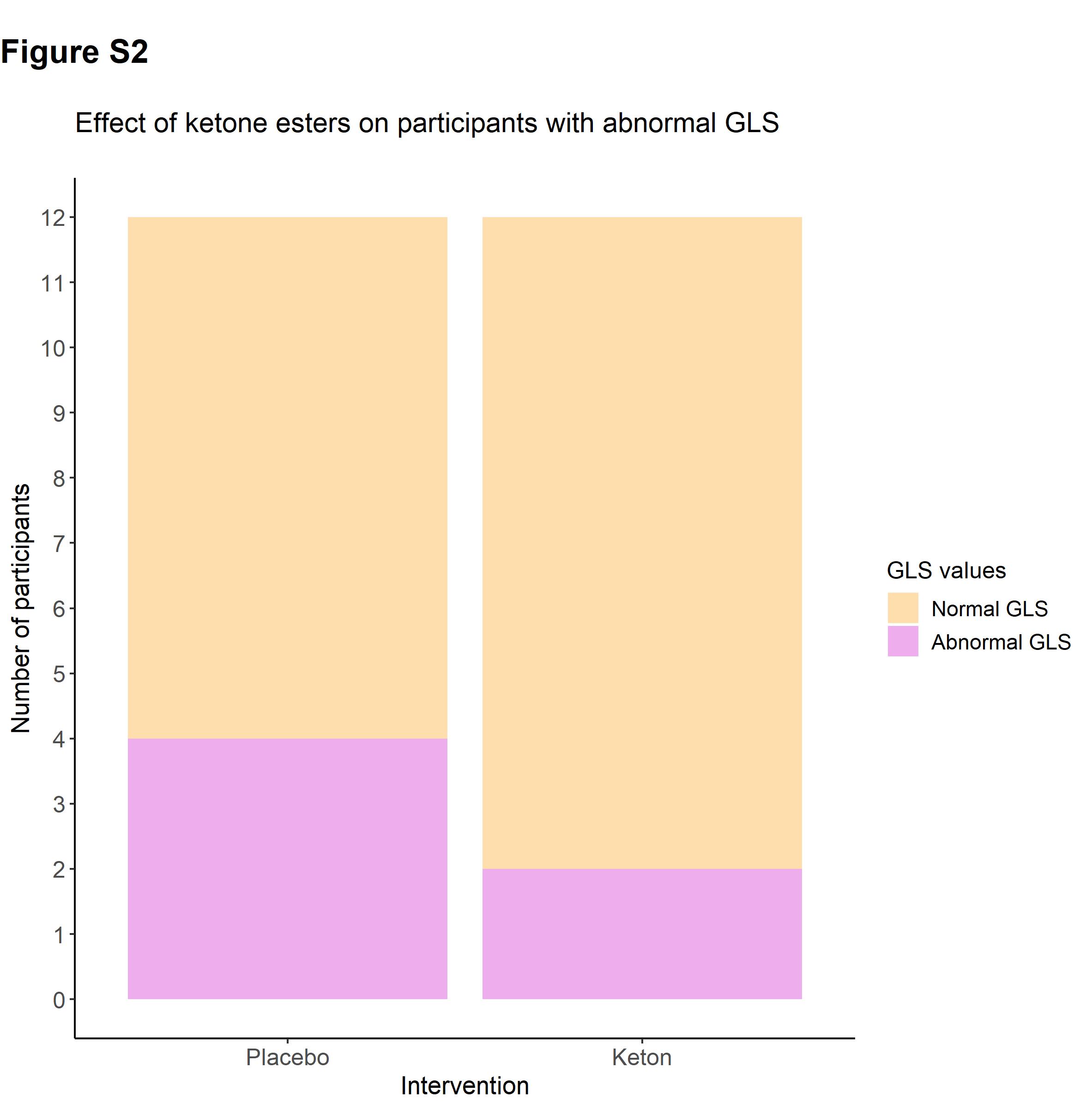

Supplement: Supplementary file 2 [file Image_2.JPEG]
