# Supplementary material for: Oral ketone esters acutely improve myocardial contractility in post-hospitalized COVID-19 patients: A randomized placebo-controlled double-blind crossover study
Source: Front Nutr. 2023 Feb 9;10:1131192. doi: 10.3389/fnut.2023.1131192 (PMC9947401; doi:10.3389/fnut.2023.1131192)
Supplement: Supplementary file 3 [file Table_1.DOCX]

**Table S1**

| **Variable** | **ECHOVID-19 follow-up** | **Present study** | **Mean difference [CI]** | ***P* value** |
| --- | --- | --- | --- | --- |
| ***Baseline characteristics*** |  |  |  |  |
| Age | 63 ± 12 | 59 ± 8 | 3 [-9;2] | p = 0.22 |
| BMI | 27.5 ± 5.8 | 27.3 ± 6.2 | 0.3 [-4.3;3.8] | p = 0.90 |
| Hypertension, n(%) | 44 (48) | 3 (25) | 0.4 [0.1;1-5]* | p = 0.22 |
| Hyperlipidaemia, n(%) | 33 (36) | 7 (58) | 2.4 [0.6;10.6]* | p = 0.21 |
| Prevalent heart failure, n(%) | 3 (3) | 0 (0) | 0.0 [0.0;19.2]* | p = 1.00 |
| Ischemic heart disease, n(%) | 7 (8) | 2 (17) | 2.4 [0.2;15.1]* | p = 0.28 |
|  |  |  |  |  |
| ***Baseline values*** |  |  |  |  |
| Systolic blood pressure (mmHg) | 126.6 ± 16.5 | 136.2 ± 14.2 | 9.6 [0.03;19.2] | p = 0.05 |
| Diastolic blood pressure (mmHg) | 82.0 ± 9.7 | 78.2 ± 7.5 | 3.8 [-2.6;10.1] | p = 0.22 |
| Heart rate (bpm) | 72.8 ± 11.1 | 61.7 ± 8.5 | -17.1 [-22.9;-11.3] | p <0.001 |
|  |  |  |  |  |
| ***Abnormal echocardiographic findings***, *n* (%) |  |  |  |  |
| Abnormal LVEF | 19 (21) | 2 (17) | 0.8 [0.1;4.0]* | p = 1.00 |
| Abnormal GLS | 27 (30) | 4 (33) | 1.2 [0.2;4.9]* | p = 0.75 |
| Abnormal TAPSE | 6 (7) | 0 (0) | 0.0 [0.0;6.8]* | p = 1.00 |

**Comparison of baseline characteristics between the present study and the ECHOVID-19 follow-up.** Data are mean ± SD or n (%). Data was analysed by t-test and two-proportion z-test. Percentage is calculated from number of measured valuables (not including missing values). Bmp = beats per minute. * indicates odds ratio with CI for having the condition in the present study.
